# Risk factor for permanent stoma and incontinence quality of life after sphincter‐preserving surgery for low rectal cancer without a diverting stoma

**DOI:** 10.1002/ags3.12033

**Published:** 2017-08-31

**Authors:** Takuya Miura, Yoshiyuki Sakamoto, Hajime Morohashi, Tatsuya Yoshida, Kentaro Sato, Kenichi Hakamada

**Affiliations:** ^1^ Department of Gastroenterological Surgery Hirosaki University Graduate School of Medicine Hirosaki Japan

**Keywords:** diverting stoma, low rectal cancer, sphincter‐preserving surgery

## Abstract

The goal of the present study was to evaluate permanent stoma formation and defecation function in long‐term follow up after surgery for low rectal cancer without a diverting stoma. Subjects were 275 patients who underwent sphincter‐preserving surgery for low rectal cancer between 2000 and 2012. Clinical outcomes were evaluated and defecation function was assessed based on a questionnaire survey, using Wexner and modified fecal incontinence quality of life (mFIQL) scores. Incidence of anastomotic leakage was 21.8%, and surgery‐related death as a result of anastomotic leakage occurred in one male patient. Median follow‐up period was 4.9 years and permanent stoma formation rate was 16.7%. Anastomotic leakage was an independent predictor of permanent stoma formation (odds ratio [OR] 5.86, *P*<0.001). Age <65 years (OR 1.99, *P*=0.001) and male gender (OR 4.36, *P*=0.026) were independent predictors of anastomotic leakage. A permanent stoma was formed as a result of poor healing of anastomotic leakage in 29.6% of males, but in no females. Defecation function was surveyed in 27 and 116 patients with and without anastomotic leakage, respectively. These groups had no significant differences in median follow‐up period (63.5 vs 63 months), Wexner scores (quartile) (6 (2.5‐9) vs 6 (3‐11)), and mFIQL scores (26.1 (4.8‐64.2) vs 23.8 (5.9‐60.7). Defecation function associated with anastomotic leakage showed no significant dependence on gender or resection procedure. Sphincter‐preserving surgery without a diverting stoma may be indicated for females with low rectal cancer. In this procedure, male gender is a risk factor for anastomotic leakage and subsequent formation of a permanent stoma in one in three patients.

## 1 INTRODUCTION

In sphincter‐preserving surgery for low rectal cancer, a diverting stoma is concomitantly formed with the aim of resting the anastomosis region until it heals.^1^, ^2^ Diverting stoma formation is recommended based on a meta‐analysis showing that this procedure reduced anastomotic leakage after low anastomosis close to the anus.^3^ However, in a multicenter study in Japan, prevention of anastomotic leakage by a diverting stoma after low anastomosis following rectal cancer resection was not found.^4^ Anal function is retained without a diverting stoma in some cases, and such patients thus undergo unnecessary stoma formation.^5^, ^6^

Our department has carried out sphincter‐preserving surgery without a diverting stoma after low anterior resection (LAR) and intersphincteric resection (ISR) for low rectal cancer as a basic treatment strategy.^6^, ^7^ The objective of this retrospective study was to investigate safety, permanent stoma formation, and defecation function in patients who underwent this procedure for low rectal cancer, and to clarify the validity and indication for this treatment.

## 2 METHODS

### 2.1 Patients

Of 370 consecutive patients with low rectal adenocarcinoma who underwent initial proctectomy at the Department of Gastroenterological Surgery, Hirosaki University, between 2000 and 2012, 298 received sphincter‐preserving surgery. Subjects of the present study were 275 of these patients, excluding one case with concomitant ulcerative colitis and 22 patients in whom a diverting stoma was formed after preoperative radiotherapy. Rectal cancer in which the lower margin was located below the peritoneal reflection during surgery was defined as low rectal cancer. Data for anastomotic leakage and perioperative complications, permanent stoma formation, and reasons for the procedure were collected from medical records. Perioperative complications were defined using the Clavien‐Dindo classification.^8^ Clinical leakage signs were defined as abdominal pain, abdominal distention, fever, and pus or fecal discharge from the pelvic drain. All clinically suspicious symptoms were confirmed by digital rectal examination and radiographic examination (e.g. extravasation of endoluminally given water‐soluble contrast enema, pelvic abscess and fluid/air bubbles surrounding the anastomosis on computed tomography).^7^ Using the proposed grading system, anastomotic leakage was classified into three grades: grade A required no active therapeutic intervention; grade B required active therapeutic intervention; and grade C required reoperation.^9^ Anastomotic leakage with grades B and C (but not grade A) within 30 days after surgery was defined as anastomotic leakage. Age, sex, body mass index (BMI), ischemic disease, diabetes, American Society of Anesthesiologists (ASA) status, intraoperative blood transfusion, tumor diameter, tumor‐anal verge distance, anastomotic height from anal verge, circumferential occupation, tumor depth, regional lymph node metastasis, distant metastasis, circumferential margin (CRM), operation time, blood loss, laparoscopy, combined resection, lateral lymph node dissection (LLND), resection procedure, and anastomosis method were examined as clinicopathological factors. When a stoma was present at final follow up, it was regarded as a permanent stoma.^10^ Median follow‐up period was 4.9 years.

### 2.2 Operative and perioperative management

In standard perioperative management, the patient fasted from the day before surgery, and received mechanical pretreatment and perioperative antibiotics before surgery and for 3 days after surgery. After pressure reduction by transanal drainage for about 1 week after surgery, food ingestion was started. After transection of the inferior mesenteric artery and vein, total mesorectal excision (TME) was carried out as a standard surgical procedure, and bilateral LLND was done when the depth was T3 or deeper, as a rule.^11^ To secure a 2‐cm resection margin, ISR was selected for tumors located within 2 cm from the upper margin of the levator ani muscle attachment region.^7^, ^8^, ^12^ For anastomosis in LAR and ISR, double‐stapled and hand‐sewn coloanal anastomosis were carried out, respectively. Side‐to‐end anastomosis was applied as a rule, and end‐to‐end anastomosis was used when the pelvis was narrow or the reconstructed intestine was short. When anastomotic leakage was clinically suspected after surgery, its presence or absence was confirmed by fluoroscopy or computed tomography (CT). If anastomotic leakage was observed, it was treated with antibiotics, a drainage tube, or stoma formation, depending on the details in each case. In approximately 6 months after stoma formation as a result of leakage, integrity of the anastomosis was checked by digital rectal examination and a water‐soluble contrast enema examination. Patients without any findings of anastomotic leakage underwent stoma closure. When findings of anastomotic leakage were sustained and the anastomosis was not expected to heal, stoma was not closed or permanent colostomy was formed. When patients had poor general condition such as unresectable distant metastases or dementia, stoma was not closed permanently even if integrity of the anastomosis was recovered.

### 2.3 Evaluation of function

Defecation function and quality of life (QOL) were surveyed using a questionnaire in patients who did and did not develop anastomotic leakage, and evaluated based on the frequency of defecation per day and the Wexner Score^13^ and modified fecal incontinence quality of life (mFIQL) score.^14^

### 2.4 Statistical analyses

Risk factors for permanent stoma formation and for anastomotic leakage were analyzed by Fisher exact test. Factors with a significant difference were subjected to multivariate logistic regression analysis. Defecation function was compared between groups by Mann‐Whitney *U*‐test. Two‐sided *P*<0.05 was regarded as significant. Statistical analysis was carried out using EZR.^15^

## 3 RESULTS

### 3.1 Background of patients and complications

Median age was 64 years old (interquartile range, IQR: 55‐71.5), 199 patients (72.4%) were male, and median BMI was 22.7 (IQR: 20.8‐24.5). Fourteen patients (5.1%) had concomitant cerebral and cardiovascular lesions, 40 (14.5%) had diabetes, 30 (10.9%) had severe complications of ASA grade 3 or 4 or higher, and nine (3.3%) received blood transfusion during surgery. Median tumor diameter was 4.5 cm (IQR: 3.0‐6.1), median tumor‐anal verge distance was 4.5 cm (IQR: 3.0‐6.0), and the tumor was circumferential in 46 cases (16.7%). Disease stage was 0 in 10 cases (3.6%), I in 72 (26.2%), II in 60 (21.8%), III in 101 (36.7%), and IV in 32 (11.6%). LAR was carried out in 157 patients (57%) and ISR was carried out in 118 (43%). Laparoscopic surgery was carried out in eight patients (2.9%), combined resection of other organs in 18 (6.5%), LLND in 167 (60.7%), and side‐to‐end anastomosis in 222 (80.7%). Median operative time was 169 (138‐238) minutes, and median blood loss was 360 mL (180‐628). CRM was positive in seven patients (2.5%). Surgery‐related death occurred in one male patient as a result of anastomotic leakage after LAR. Clavien‐Dindo classification was III or higher in 72 cases (26.2%), and respiratory or dialysis management in an intensive care unit was necessary in six (2.2%). Anastomotic leakage of grades B and C occurred in 30 patients each (rates of 10.9% each) and the overall incidence was 21.8% (60/275) (Table 1).

**Table 1:** Clinicopathological characteristics of 275 patients who underwent sphincter‐preserving surgery for low rectal cancer between 2000 and 2012

Variable	Value
Age (y)	64 (55‐71.5)
Gender, n (%)	
Male	199 (72.4)
Female	76 (27.6)
Body mass index (kg/m^2^)^a^	22.7 (20.8‐24.5)
Ischemic disease, n (%)	14 (5.1)
Diabetes, n (%)	40 (14.5)
ASA 3‐4, n (%)	30 (10.9)
Blood transfusion, n (%)	9 (3.3)
Tumor size (cm)^a^	4.5 (3.0‐6.1)
Distance from anal verge to tumor (cm)^a^	4.5 (3.0‐6.0)
Anastomotic height from anal verge (cm)^a^	3.0 (1.5‐4.0)
Circumferential occupation, n (%)	46 (16.7)
Pathological TNM stage, n (%)	
0	10 (3.6)
I	72 (26.2)
II	60 (21.8)
III	101 (36.7)
IV	32 (11.6)
Type of resection, n (%)	
Low anterior resection	157 (57.1)
Intersphincteric resection	118 (42.9)
Laparoscope‐assisted surgery, n (%)	8 (2.9)
Combined resection, n (%)	18 (6.5)
Lateral lymph node dissection, n (%)	167 (60.7)
Side to end anastomosis, n (%)	222 (80.7)
Operation time (min)^a^	169 (138‐238)
Blood loss (mL)^a^	360 (180‐628)
CRM positive, n (%)	7 (2.5)
Complications (Clavien‐Dindo), n (%)	
All (I‐V)	135 (49.1)
III	66 (24.0)
IV	6 (2.2)
V	1 (0.45)
Anastomotic leakage, n (%)	
Grade B	30 (10.9)
Grade C	30 (10.9)

a Median (interquartile range).

ASA, American Society of Anesthesiologists; CRM, circumferential margin.

### 3.2 Risk factors for permanent stoma formation

**Figure 1:**
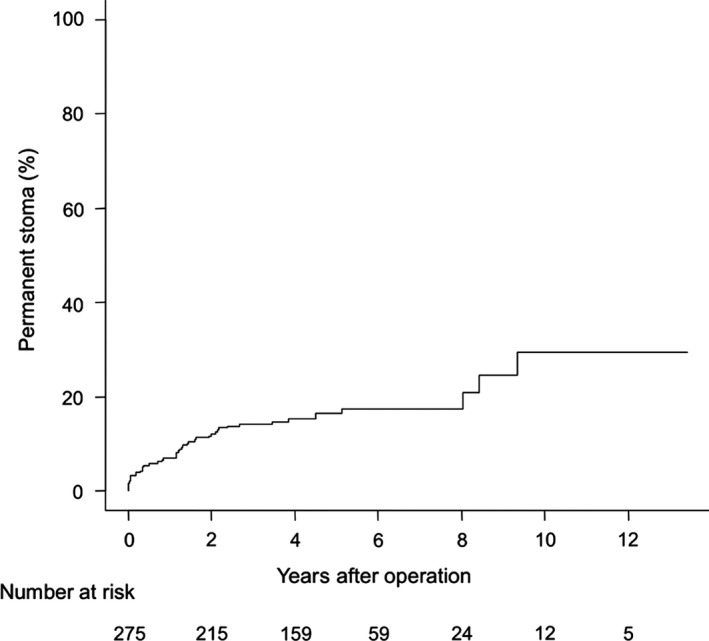
Incidence of permanent stoma formation after sphincter‐preserving surgery without a diverting stoma for low rectal cancer

Five‐year cumulative permanent stoma formation rate was 16.7% in a median follow‐up period of 4.9 years (Figure 1). Reason for permanent stoma was poor healing of anastomotic leakage in 16 patients (35.5%), metastatic disease after anastomotic leakage in three (6.7%), delayed onset of anastomotic leakage in four (8.9%), local recurrence in 19 (42.2%), poor defecation function in two (4.4%), and perforation of sigmoid colon as a result of radiation therapy for bone metastasis in one (2.2%). Delayed onset of anastomotic leakage occurred between 2 months and 8 years after surgery. All resulted in the status of permanent stoma because of dementia in one male and one female, metastatic disease in one man, and poor healing of anastomotic leakage in one male. In univariate analysis, tumor‐anal verge distance <5 cm, ISR, end‐to‐end anastomosis, and anastomotic leakage were identified as significant risk factors, and anastomotic leakage was an independent risk factor for permanent stoma formation in multivariate analysis (odds ratio (OR): 5.86, *P*<0.001) (Table 2).

**Table 2:** Univariate and multivariate analyses of the dependence of permanent stoma on clinicopathological variables

Variables	n	Univariate analysis		Multivariate analysis	
		n (%)	*P* value	Odds ratio (95% CI)	*P* value
Age (y)					
≥65	132	16 (12.1)	0.074		
<65	143	29 (20.3)			
Gender					
Female	76	10 (13.2)	0.467		
Male	199	35 (17.6)			
Body mass index (kg/m^2^)					
<25	214	35 (16.4)	1		
≥25	61	10 (16.4)			
Ischemic disease					
No	261	43 (16.5)	1		
Yes	14	2 (14.3)			
Diabetes					
No	235	40 (17.0)	0.644		
Yes	40	5 (12.5)			
ASA					
1, 2	245	40 (16.3)	1		
3, 4	30	5 (16.7)			
Blood transfusion					
No	266	42 (15.8)	0.168		
Yes	9	3 (33.3)			
Tumor size (cm)					
<5	157	20 (12.7)	0.070		
≥5	118	25 (21.2)			
Tumor location from AV (cm)					
≥5	137	16 (11.7)	0.049	1.98 (0,86‐4.53)	0.107
<5	138	29 (21.0)			
Anastomotic height from AV (cm)					
>4	56	5 (8.9)	0.107		
≤4	219	40 (18.3)			
Circumferential occupation					
No	229	36 (15.7)	0.516		
Yes	46	9 (19.6)			
Tumor depth					
T1, T2	106	15 (14.2)	0.504		
T3, T4	169	30 (17.8)			
Regional lymph node metastasis					
No	146	21 (14.4)	0.415		
Yes	129	24 (18.6)			
Distant metastasis					
No	243	42 (17.3)	0.318		
Yes	32	3 (9.4)			
CRM					
Negative	268	44 (16.4)	1		
Positive	7	1 (14.3)			
Operation time (min)					
<160	113	18 (15.9)	1		
≥160	162	27 (16.7)			
Blood loss (mL)					
<360	136	21 (15.4)	0.746		
≥360	139	24 (17.3)			
Laparoscopy					
No	267	44 (16.5)	1		
Yes	8	1 (12.5)			
Combined resection					
No	257	39 (15.2)	0.09		
Yes	18	6 (33.3)			
Lateral lymph node dissection					
No	108	12 (11.1)	0.067		
Yes	167	33 (19.8)			
Type of resection					
LAR	157	19 (12.1)	0.032	1.43 (0.56‐3.63)	0.456
ISR	118	26 (22.0)			
Side to end anastomosis					
No	53	15 (28.3)	0.012	0.62 (0.24‐1.60)	0.332
Yes	222	30 (13.5)			
Anastomotic leakage					
No	215	23 (10.7)	<0.001	5.86 (2.82‐12.20)	<0.001
Yes	60	22 (36.7)			

ASA, American Society of Anesthesiologists; AV, distance from anal verge to tumor; CI, confidence interval; CRM, circumferential resection margin; ISR, intersphincteric resection; LAR, low anterior resection.

### 3.3 Risk factors for anastomotic leakage

Age <65 years old and male gender were significant risk factors for anastomotic leakage in univariate analysis, and were also independent risk factors in multivariate analysis (age <65 years old: OR=1.99, *P*=0.001; male: OR=4.36, *P*=0.026) (Table 3). After development of anastomotic leakage, a permanent stoma was formed as a result of poor healing of anastomotic leakage in 29.6% of males (about one in three patients), regardless of age. In contrast, no permanent stoma as a result of poor healing of anastomotic leakage formed in females, regardless of age (Table 4).

**Table 3:** Univariate and multivariate analyses of the dependence of anastomotic leakage on clinicopathological variables

Variables	n	Univariate analysis		Multivariate analysis	
		n (%)	*P* value	Odds ratio (95% CI)	*P* value
Age					
≥65	132	21 (15.9)	0.028	1.99 (1.09‐3.65)	0.026
<65	143	39 (27.3)			
Gender					
Female	76	6 (7.9)	<0.001	4.36 (1.78‐10.70)	0.001
Male	199	54 (27.1)			
Body mass index (kg/m^2^)					
<25	214	41 (19.2)	0.053		
≥25	61	19 (31.1)			
Ischemic disease					
No	261	57 (21.8)	1		
Yes	14	3 (21.4)			
Diabetes					
No	235	48 (20.4)	0.213		
Yes	40	12 (30.0)			
ASA					
1, 2	245	51 (20.8)	0.248		
3, 4	30	9 (30.0)			
Blood transfusion					
No	266	58 (21.8)	1		
Yes	9	2 (22.2)			
Tumor size (cm)					
<5	157	30 (19.1)	0.239		
≥5	118	30 (25.4)			
Tumor location from AV (cm)					
≥5	137	36 (26.3)	0.081		
<5	138	24 (17,4)			
Anastomotic height from AV (cm)					
>4	56	12 (21.4)	1		
≤4	219	48 (21.9)			
Circumferential occupation					
No	229	48 (21.0)	0.439		
Yes	46	12 (26.1)			
Tumor depth					
T1, T2	106	22 (20.8)	0.766		
T3, T4	169	38 (22.5)			
Regional lymph node metastasis					
No	146	30 (20.5)	0.661		
Yes	129	30 (23.3)			
Distant metastasis					
No	243	54 (22.2)	0.821		
Yes	32	6 (18.8)			
CRM					
Negative	268	59 (22.0)	1		
Positive	7	1 (14.3)			
Operation time (min)					
<160	113	29 (25.7)	0.235		
≥160	162	31 (19.1)			
Blood loss (mL)					
<360	136	27 (19.9)	0.468		
≥360	139	33 (23.7)			
Laparoscopy					
No	267	60 (22.5)	0.207		
Yes	8	0 (0.0)			
Combined resection					
No	257	57 (22.2)	0.771		
Yes	18	3 (16.7)			
Lateral lymph node dissection					
No	108	25 (23.1)	0.765		
Yes	167	35 (21.0)			
Type of resection					
LAR	157	38 (24.2)	0.303		
ISR	118	22 (18.6)			
Side‐to‐end anastomosis					
No	53	13 (24.5)	0.583		
Yes	222	47 (21.2)			

ASA, American Society of Anesthesiologists; AV, distance from anal verge to tumor; CI, confidence interval; CRM, circumferential resection margin; ISR, intersphincteric resection; LAR, low anterior resection.

**Table 4:** Permanent stoma as a result of poor healing of leakage stratified by risk factors for anastomotic leakage and type of resection

	n	Anastomotic leakage, n (%)	Permanent stoma as a result of poor healing of leakage, n (%)^a^
Male	199	54 (27.1)	16 (29.6)
≥65 y	94	19 (20.2)	6 (31.6)
<65 y	105	35 (33.3)	10 (28.6)
LAR	113	33 (29.2)	9 (27.3)
ISR	86	21 (24.4)	7 (33.3)
Female	76	6 (7.8)	0 (0)
≥65 y	38	2 (5.3)	0 (0)
<65 y	38	4 (10.5)	0 (0)
LAR	44	5 (11.4)	0 (0)
ISR	32	1 (3.1)	0 (0)

a Rate in patients with anastomotic leakage.

ISR, intersphincteric resection; LAR, low anterior resection.

### 3.4 Defecation function and QOL

Defecation function was surveyed by mailing a questionnaire to 27 patients who developed anastomotic leakage (response rate: 45.0%) and 116 patients who did not develop anastomotic leakage (response rate: 53.9%). Frequency of defecation per day and Wexner and mFIQL scores were compared for these groups. Comparing the backgrounds of the two groups, the anastomotic leakage group had a significantly higher rate of BMI ≥25 kg/m^2^ (44.4% vs 23.3%), but the median follow‐up periods of 63.5 and 63 months, respectively, and all other clinicopathological factors did not differ significantly between the groups. Median (quartile) frequency of defecation per day and Wexner and mFIQL scores were 4 (1.5‐4), 6 (2.5‐9), and 26.1 (4.8‐64.2), respectively, in patients with anastomotic leakage, and 4 (1.5‐4), 6 (3‐11), and 23.8 (5.9‐60.7), respectively, in those without anastomotic leakage, with no significant differences between the groups (Table 5). Gender and resection procedure had no significant effect on anastomotic leakage‐associated defecation function or QOL (Table 5).

**Table 5:** Defecation function and QOL stratified by anastomotic leakage

Responders	Anastomotic leakage	No anastomotic leakage	*P* value
All responders, n (%)	27 (45.0)^a^	116 (53.9)^a^	
Period (mo)	63.5 (43.7‐79.7)	63.0 (48.0‐94.0)	0.582
Bowel movements	4.0 (1.5‐4.0)	4.0 (1.5‐4.0)	0.641
Wexner score	6.0 (2.5‐9.0)	6.0 (3.0‐11.0)	0.513
mFIQL score	26.1 (4.8‐64.2)	23.8 (5.9‐60.7)	0.719
Male responders, n (%)	21 (38.8)^a^	75 (43.6)^a^	
Bowel movements	4.0 (4.0‐4.0)	4.0 (1.5‐4.5)	0.556
Wexner score	5.0 (2.0‐12.0)	8.0 (3.0‐12.0)	0.256
mFIQL score	26.2 (7.1‐57.1)	33.3 (9.5‐65.5)	0.585
Female responders, n (%)	6 (100)^a^	41 (58.5)^a^	
Bowel movements	1.0 (0.5‐5.6)	4.0 (0.5‐4.0)	0.608
Wexner score	6.5 (6.0‐8.5)	4.0 (2.0‐8.0)	0.424
mFIQL score	14.8 (2.9‐56.2)	11.9 (4.8‐38.1)	0.86
LAR responders, n (%)	16 (42.1)^a^	68 (57.1)^a^	
Bowel movements	4.0 (3.3‐4.0)	4.0 (1.3‐4.0)	0.32
Wexner score	4.0 (0.0‐7.5)	4.5 (2.0‐8.0)	0.482
mFIQL score	14.8 (3.6‐39.8)	23.8 (6.5‐43.4)	1
ISR responders, n (%)	11 (50.0)^a^	48 (50.0)^a^	
Bowel movements	4.0 (1.2‐5.5)	4.0 (1.5‐7.0)	0.796
Wexner score	7.0 (5.5‐14.0)	9.0 (4.7‐14.0)	0.711
mFIQL score	50.0 (16.6‐69.0)	36.9 (4.8‐74.4)	1

Data are shown as median (interquartile range).

a % response rate in the respective group.

ISR, intersphincteric resection; LAR, low anterior resection; mFIQL, modified fecal incontinence quality of life; QOL, quality of life.

## 4 DISCUSSION

A diverting stoma may contribute to prevention of anastomotic leakage in cases with low anastomosis near the anus, and is generally formed in anus‐preserving surgery for low rectal cancer.^1^, ^2^, ^3^ However, in a recent multicenter study in Japan, a diverting stoma did not reduce the incidence of anastomotic leakage after low anastomosis, but did significantly reduce the rate of reoperation after anastomotic leakage.^4^ There was no difference in mortality between patients with and without diverting stoma formation,^4^ suggesting that a diverting stoma is unnecessary if anastomotic leakage is treated appropriately, including with reoperation.

As complications associated with diverting stoma formation and stoma closure may develop, this procedure is not necessarily a safe intervention.^16^ Therefore, if a diverting stoma does not reduce anastomotic leakage‐ and surgery‐related deaths, patients who are unlikely to develop anastomotic leakage undergo an unnecessary and risky procedure. Therefore, the significance of a diverting stoma requires investigation, including the rate of permanent stoma formation and defecation function. The significance of the present study is that the indication for diverting stoma was investigated based on long‐term anal conditions, defecation function, and QOL as outcomes.

One of the main goals was to identify the perioperative risk factors for permanent stoma in all aspects in consecutive patients with low rectal cancer at a tertiary hospital. We considered that this analysis could offer valuable overview of the consequences after sphincter‐preserving surgery for low rectal cancer without a diverting stoma to patients and physicians. We first identified anastomotic leakage as a risk factor for permanent stoma formation, as previously found.^17^, ^18^, ^19^, ^20^, ^21^ Age <65 years and male gender were then identified as independent risk factors for anastomotic leakage. A permanent stoma was formed as a result of poor healing of anastomotic leakage in one in three males, regardless of age and resection procedure. In contrast, in females, the incidence of anastomotic leakage was low and there was no permanent stoma formation as a result of poor healing of anastomotic leakage.

Reduction of defecation function is of concern when anastomotic leakage occurs, but findings have varied among previous studies.^22^, ^23^, ^24^ In our patients, anastomotic leakage did not contribute to reduction of long‐term defecation function and QOL, and there was no influence of sex or resection procedure. Thus, in female patients, sphincter‐preserving surgery for low rectal cancer without diverting stoma formation is unlikely to have a negative influence on permanent stoma formation as a result of poor healing of anastomotic leakage and reduced long‐term defecation function and QOL. In a study of short‐term anastomotic leakage following diverting stoma formation after low anastomosis, a diverting stoma was found to be useful in males, but not in females.^25^ The long‐term anal function in our study supports the validity of low anastomosis without diverting stoma formation after rectal resection in female patients. As there is no clear basis for expecting long‐term improvement of defecation function and QOL by accepting reduction of QOL as a result of diverting stoma formation, a diverting stoma should be formed only in cases in which it is likely to be beneficial.

The incidence of anastomotic leakage was high in our patients, but mortality was low, suggesting that appropriate treatment was carried out. However, strategies to prevent anastomotic leakage are necessary.^26^ Many factors influence anastomotic leakage, but male gender is a common risk factor in many reports, but no specific countermeasures for males have been developed.^2^, ^4^, ^25^, ^27^, ^28^, ^29^ Diverting stoma formation has been proposed, but it is uncertain if this approach reduces the incidence of anastomotic leakage.^4^, ^25^ A low incidence of anastomotic leakage has been reported in laparoscopic surgery, and we have introduced laparoscopic rectal resection and attempted to improve the quality of the operation by pursuing the Japan Society for Endoscopic Surgery (JSES) technical qualifications. Reduction of the incidence of anastomotic leakage is expected with improvement of the surgical technique,^30^ but male gender is still a risk factor for anastomotic leakage after laparoscopic surgery.^29^, ^31^ In addition to the improvement of surgical quality, a new strategy such as transanal approach with intraoperative blood perfusion assessment might offer safe sphincter‐preserving surgery to patients at high risk of anastomotic leakage.^32^, ^33^ Given the high incidence of anastomotic leakage and high rate of permanent stoma formation as a result of poor healing after anastomotic leakage in male patients in the current study, it may be desirable to form a diverting stoma in males until identification of reliable predictors of a low risk of anastomotic leakage after low anastomosis. For patients with risk factors of local recurrence, the introduction of preoperative adjuvant therapy and precise assessment of tumor extension by high‐resolution magnetic resonance imaging (MRI) could offer oncological safe sphincter‐preserving surgery and justify inevitable abdominoperineal resection.^34^

There are several limitations in the present study. First, it was carried out as a single‐center retrospective observational study, and only a small number of patients treated with currently accepted laparoscopic surgery were included. The high rate of anastomotic leakage in this single center suggested that there might have been some technical problems which could be a limitation of this study. Second, only about 50% of patients responded to the questionnaire on evaluation of anal function, and the presence of a bias as a result of non‐respondents cannot be ruled out. Third, the subjects were patients who did not receive preoperative treatment. Such treatment for low rectal cancer is not specified as a standard approach in current Japanese guidelines, but preoperative chemoradiotherapy is standard treatment in Western countries.^35^ Our institution has formed a diverting stoma in patients treated with preoperative radiotherapy based on poor healing of the anastomosis region, and a diverting stoma may be significant for prevention of anastomotic leakage in female patients treated with preoperative radiotherapy.^36^ In contrast, favorable local outcomes have been reported in Europe for rectal cancer treated with surgery alone without preoperative treatment, with selection of patients based on preoperative high‐resolution MRI.^37^ Patients similar to the subjects in the current study are likely to increase worldwide, and thus our results may be significant, despite the above limitations.

## 5 CONCLUSIONS

Sphincter‐preserving surgery for low rectal cancer without diverting stoma formation may be indicated for female patients. Male gender was a risk factor for anastomotic leakage in this procedure, with a permanent stoma as a result of anastomotic leakage formed in one in three male patients.

## DISCLOSURE

The protocol for this research project has been approved by a suitably constituted Ethics Committee of the institution and conforms to the provisions of the Declaration of Helsinki. The study was approved by the Ethics Committee of our institution (2016‐1006, 2016‐1033).

Conflict of Interest: Authors declare no conflicts of interest for this article.
